# NAT10 Maintains *OGA* mRNA Stability Through ac4C Modification in Regulating Oocyte Maturation

**DOI:** 10.3389/fendo.2022.907286

**Published:** 2022-07-22

**Authors:** Jiayu Lin, Yuting Xiang, Jiana Huang, Haitao Zeng, Yanyan Zeng, Jiawen Liu, Taibao Wu, Qiqi Liang, Xiaoyan Liang, Jingjie Li, Chuanchuan Zhou

**Affiliations:** ^1^ Reproductive Medicine Center, Sixth Affiliated Hospital of Sun Yat-sen University, Guangzhou, China; ^2^ Department of Obstetrics and Gynecology, Affiliated Dongguan People’s Hospital, Southern Medical University, Dongguan, China

**Keywords:** oocyte, *in vitro* maturation, NAT10, N4-acetylcytidine, OGA, O-GlcNAc, transcription

## Abstract

*In vitro* maturation (IVM) refers to the process of developing immature oocytes into the mature *in vitro* under the microenvironment analogous to follicle fluid. It is an important technique for patients with polycystic ovary syndrome and, especially, those young patients with the need of fertility preservation. However, as the mechanisms of oocyte maturation have not been fully understood yet, the cultivation efficiency of IVM is not satisfactory. It was confirmed in our previous study that oocyte maturation was impaired after N-acetyltransferase 10 (NAT10) knockdown (KD). In the present study, we further explored the transcriptome alteration of NAT10-depleted oocytes and found that *O-GlcNAcase*(*OGA*) was an important target gene for NAT10-mediated ac4C modification in oocyte maturation. NAT10 might regulate *OGA* stability and expression by suppressing its degradation. To find out whether the influence of NAT10-mediated ac4C on oocyte maturation was mediated by *OGA*, we further explored the role of *OGA* in IVM. After knocking down OGA of oocytes, oocyte maturation was inhibited. In addition, as oocytes matured, *OGA* expression increased and, conversely, O-linked N-acetylglucosamine (O-GlcNAc) level decreased. On the basis of NAT10 KD transcriptome and OGA KD transcriptome data, NAT10-mediated ac4C modification of *OGA* might play a role through G protein–coupled receptors, molecular transduction, nucleosome DNA binding, and other mechanisms in oocyte maturation. *Rsph6a*, *Gm7788*, *Gm41780*, *Trpc7*, *Gm29036*, and *Gm47144* were potential downstream genes. In conclusion, NAT10 maintained the stability of *OGA* transcript by ac4C modification on it, thus positively regulating IVM. Moreover, our study revealed the regulation mechanisms of oocytes maturation and provided reference for improving IVM outcomes. At the same time, the interaction between mRNA ac4C modification and protein O-GlcNAc modification was found for the first time, which enriched the regulation network of oocyte maturation.

## Introduction

*In vitro* oocyte maturation (IVM) is a promising assisted reproductive technology simulating the physiological development of oocytes from the immature, also called germinal vesicle (GV) stage, to the mature, which was also called metaphase II (MII) (1, 2). IVM presents great clinical application values, especially for those patients with indications for fertility preservation requirement, with polycystic ovary syndrome or high risk of ovarian hyperstimulation syndrome, etc. (3, 4). However, because the underlying mechanisms involved in oocyte maturation have not been fully understood, IVM has not achieved satisfying clinical outcomes compared with conventional *in vitro* fertilization and other assisted reproductive techniques. Therefore, it is very necessary to explore the mechanisms of IVM, which will help to discover new molecular targets and directions for improving its clinical application.

The process of oocyte maturation is temporally and spatially monitored to permit the proper and accurate expression of genes, which is highly dependent upon post-transcriptional regulation of messenger RNA (mRNA) (5, 6). In early gametes, it is mainly achieved through epigenetic mechanisms, which is necessary for ovulation and zygote to build up competence during the maternal-to-zygotic genome transition (MZT) (7, 8). Therefore, the role of epigenetic modifications is crucial, and the underlying mechanisms remain to be further explored.

More than 100 epigenetic modifications of mRNA, including N6-adenosine methylation (m6A), cytosine hydroxylation (m5C), and N1-adenosine methylation (m1A), have been revealed in mediating the stability, function, and splicing process of targeted mRNAs (9–11). Previous studies on m6A have reported that two important readers, YTHDF2 and YTHDC1, were respectively located in the nucleus and cytoplasm of oocytes, which played crucial roles in mRNA degradation, transcriptome switching, and selective splicing during oocyte maturation. KIAA1429 is a recently identified component of the m6A writers, affecting the quality of oocytes by mediating meiosis, chromatin remodeling, and selective splicing of genes associated with oogenesis (12).

N4-acetylcytidine (ac4C) is the first acetylcytidine event and highly conserved epigenetic modification in eukaryotic mRNAs reported in recent years (13, 14). Enriched in the coding sequence (CDS) region of genes, it gradually decreases along the 5′ end to the 3′ end of the aimed transcripts (15). NAT10, as the only identified acetyltransferase, is thought to play critical roles in promoting mRNA stability and maintaining translation fidelity through ac4C modification on the specific sequence of target mRNAs (16). The dysregulation of NAT10 inhibited cell development progress and led to various diseases, such as gastric cancer and systemic lupus erythematosus (17–19).

Our team has previously demonstrated that NAT10-mediated ac4C modification affected oocyte maturation. In the NAT10 KD group, GV stage oocytes could not reach a satisfying first polar body extrusion rate compared with the negative control one (20). However, related downstream genes and potential mechanisms underneath it remain unclear. By analyzing transcriptomic data of the NAT10-depleted oocytes, *OGA* (also known as *Mgea5*) was verified as one of the important targets of NAT10-mediated ac4C modification in regulating oocyte maturation. OGA depletion caused impaired oocyte maturation, which resulted in the fluctuation in O-GlcNAc in IVM. NAT10 might regulate the stability of *OGA* transcript by ac4C modification on it, which further mediated oocyte maturation through G protein–coupled receptors, molecular transduction, and nucleosome DNA binding. *Rsph6a*, *Gm7788*, *Gm41780*, *Trpc7*, *Gm29036*, and *Gm*47144 were identified as candidate downstream genes.

Our study aimed to investigate the mechanisms of NAT10-mediated ac4C during IVM and further explored the role of downstream gene *OGA* in oocyte maturation. As OGA is an important regulatory enzyme of O-GlcNAc modification, our research will provide reference for understanding the interaction between ac4C modification and O-GlcNAcylation.

## Materials and Methods

### Mice

Three- to 4-week-old female C57BL/6 mice were purchased from Vital River Laboratory Animal Technology Co., Ltd (Beijing, China). The mice were housed in the animal laboratory center with a controlled 12-h:12-h light/dark cycle, humidity (50%–60%), and temperature (22°C–24°C). Before experiments, there was 1 week for the mice to adapt to the environment. Water and food were freely accessed to. In addition, all the interventions were approved by the Animal Care and Use Committee of the Sixth Affiliated Hospital, Sun Yat-sen University (Guangzhou, China) (ethical approval number: IACUC-2021112502).

### Oocytes Collection

Female C57BL/6 mice were intraperitoneally injected with 10 International Units of pregnant mare’s serum gonadotropin (PMSG) (Ningbo Second Hormone Factory, Zhejiang, China) 46–48 h earlier. The ovaries were then dissected and obtained. After the fat around the ovarian tissue being removed, the antral follicles were punctured with a sterile needle in a petri dish containing M2 medium (Sigma-Aldrich, M7167) and cumulus-oocyte complexes (COCs) were released.

### Oocyte *In Vitro* Maturation

The IVM medium was made from TCM-199 (Gibco, 31100035), 0.2 mM sodium pyruvate, and 10% fetal bovine serum (FBS). GV oocytes were cultured in IVM medium in a cell incubator of 5% CO_2_ at 37°C for 14–16 h. Then, GV oocytes were isolated gently from COCs in the hyaluronidase (Sigma-Aldrich, 37326-33-3) by repeatedly pipetting. The oocytes were collected for maturation rates calculation and further analyses.

### NAT10/OGA Knockdown by Trim-Away and Electroporation

Trim-Away is a newly discovered degradation method that recruits proteasome to hydrolysis antibody-bound proteins through *Trim 21* mRNA (21). Because of its high specificity, Trim-Away has been wildly used in oocytes and embryos (22, 23). To explore the effect of NAT10-mediated ac4C modification on *OGA*, *OGA* on oocyte maturation, and further on O-GlcNAc modification, we conducted targeted degradation of endogenous NAT10 and OGA in oocytes based on electroporation and Trim-Away. NAT10 antibody (ProteinTech, 13365-1-AP) and OGA antibody (ProteinTech, 14711-1-AP) were purified in advance to reduce harmful chemicals intervening in oocyte maturation such as sodium azide. First, 20 μl of antibody was pipetted into an Eppendorf tube and then 180 μl of phosphate-buffered saline (PBS) was added to dilute the antibody. Ultrafiltration tube (Millipore, UFC5100BK) was used to concentrate the antibody at 14,000 g for 10 min. The filtrate was discarded, and the inner tube was placed into a new Eppendorf tube invertedly. After centrifugation at 1,000 g for 2 min, the antibody was collected and prepared. Later, denuded GV oocytes were placed in the Tyrode’s solution (Leagene, CZ0060) for 10 s to weaken the zona pellucida. Then, they were washed for three times in Opti-MEM medium to reduce the Tyrode’s solution as much as possible. In addition, the oocytes were transferred to the antibody-containing Opti-MEM medium (total volume of 5 μl), which were then transferred into the electrode groove and waited to be electroporated. The electroporation procedure was executed according to what we have reported (1-ms pulse width, 30 volts in amplitude, and 4 pulses at intervals of 50 ms) (20). For the experimental group, we electroporated *Trim 21* mRNA and NAT10 antibody or OGA antibody in Opti-MEM medium at a final concentration of 200 ng/μl into GV stage oocytes. As a control, homologous IgG (Fine Test, PNSA-0106) and *Trim 21* mRNA were delivered into at the same concentration. Afterward, the oocytes were washed for three times and incubated in Opti-MEM medium to recover for 30 min. Then, the oocytes would be transferred to IVM medium for evaluating maturation rates 14–16 h later or transferred to a cell incubator with 3-isobutyl-1-methyl-xanthine (IBMX)–containing IVM medium (50 μM IBMX) to be kept arrested at GV stage and waited for the adequate degradation of the aimed proteins until immunofluorescence. The IBMX (HY-12318) was purchased from MCE, Shanghai, China.

### Fluorescent-Labeled Antibody Technique

To avoid the combination of the secondary antibodies in immunofluorescence with the antibodies used in electroporation, the OGA antibody was labeled with 647 fluorescence, the NAT10 antibody with 488 fluorescence, and the OGT antibody (CST, D1D8Q) with 555 fluorescence. The experiments were performed almost according to the manufacturer’s instructions of LinKine AbFluorTM 647/488/555 Labeling Kit (LinKine, KTL0560; LinKine, KTL0520; Linkine, KTL0530). To get the optimal labeling effect, the unlabeled antibody should be purified in advance. In addition, the final concentration should reach 2 mg/ml. Then, 1 μl of AbFluorTM 647/488/555 labeling solution was added to the 20 μl of the aimed antibody and gently mixed with a pipette. Activated AbFluorTM 647/488/555 solution (0.5 μl) was later added into, mixed evenly, and incubated in 37°C under the dark for 1 h. Centrifuged at 12,000 g, 4°C for 20 min, the supernatant was collected and the filtrate was discarded. PBS (30 μl) was added into and, after being mixed evenly, the liquid was centrifuged at 12,000 g, 4°C for another 20 min. The purification column was then taken out and upturned into a new a clean centrifugal tube. Then, it was centrifuged for the last 2 min, 4,000 g, 4°C. The solution collected from the centrifugal tube was the final labeled antibody.

### Immunofluorescence Staining of Oocytes

The oocytes were fixed in 1% paraformaldehyde and 0.2% Triton X-100 in PBS for 1 h at room temperature. After 1 h, the oocytes were transferred into 3% bovine serum albumin (BSA) in PBS to be blocked for another 1 h. Next, the oocytes were incubated with fluorescent-labeled antibodies against OGA (1:200), NAT10 (1:200), O-GlcNAc Transferase (OGT) (1:200), and/or another first antibody RL2 (1:200, Abcam, ab2739) at 4°C overnight. After three washes with 0.3% BSA, oocytes incubated with RL2 first antibody were then incubated with Cy3-conjugated secondary antibody (1:500, Earthox, E031620) at room temperature for 1 h in the dark condition. The oocytes were then washed with 0.3% BSA for three times. In addition, images were taken under the inverted phase contrast confocal microscope (LSM 880, Zeiss, JENA, Germany).

### NAT10/ac4C RNA Immunoprecipitation

Human embryonic kidney HEK293 cells (FuHeng Biology, FH0244) were cultured in high-glucose DMEM medium (Gibco, C11960500BT) supplemented with 10% FBS and 1% penicillin-streptomycin, at 37°C and 5% CO_2_ in a humidified atmosphere. According to manufacturer’s instructions of PEI Transfection Reagent (ProteinTech, PR40001), the NAT10-overexpressing plasmids (GeneCopeia, EX-I5674-M11) were transfected into HEK293 cells. After 48 h, the NAT10-overexpressed HEK293 cells were washed by cold PBS, mechanically isolated with a cell scraper, and centrifuged at 1,500 rpm for 5 min at 4°C. The supernatant was discarded, and the sediment was resuspended by 1 ml of purification buffer, 0.5% NP40, and 1% protease inhibitor (APExBIO, K1007). The mixture was pre-cooled in ice for 5 min and then transferred to −80°C for more than 15 min. Then, it was centrifuged at 12,000 g, 4°C for 10 min. Protein A/G magnetic beads (MCE, HY-K0202) were activated with purification buffer and conjugated with 5 μg of polyclonal anti-NAT10 antibody, 5 μg of anti-ac4C antibody (Abcam, ab252215), and 5 μg of rabbit IgG antibody (FineTest, FNSA-0106) at room temperature for 2 h, respectively. After that, the beads were washed with purification buffer for two to three times. In addition, 10% of the cell lysate was saved as input at -80°C. Antibody-conjugated A/G magnetic beads were incubated with 45% of cell lysate, 0.25 M EDTA, and 1 μl RNase inhibitor (APExBIO, K1046) at 4°C for 4 h separately. Then, the beads were washed with purification buffer for another two to three times, and all the solution was discarded. Later, the A/G magnetic beads were incubated with 117 μl of lysis buffer, 15 μl of 10% SDS, and 18 ul proteinase K of 10 mg/ml at 55°C for 30 min to purify mRNA. Moreover the reverse transcription was performed, and PCR was followed to test the binding of target RNA.

### RNA Degradation Test, RNA Extraction, and Quantitative Real-Time PCR

Human embryonic kidney HEK293 cells were firstly seeded in six wells in 24-well plates (4 × 10^5^ cells per well). They were cultured in high-glucose DMEM medium containing 10% FBS and 1% penicillin-streptomycin at 37°C and 5% CO_2_ in humidified atmosphere. According to manufacturer’s instructions of PEI Transfection Reagent, the NAT10-overexpressing plasmids were transfected into HEK293 cells in the experimental group, and the control group cells were transfected with no-load plasmids. After 48 h, the cells were exposed to Actinomycin D (5 μg/ml, Aladdin, A113142) to block RNA synthesis as previously described (24). In addition, HEK293 cells were then harvested at 0, 2, and 12 h, respectively. The total RNA of cells was then extracted and reversely transcribed using the RNeasy Micro Kit (Qiagen, 74004) and HiScript III RT SuperMix for Quantitative PCR (qPCR) (Vazyme, R323-01) according to the manufacturer’s instructions. RealStar Green Power Mixture (2×) (Genstar, A311-101) was used to carry out qPCR on the Roche LightCycler 480 II (Roche Diagnostics, Germany). The expression levels of *OGA* mRNA in the experimental group and the control group at 0 h were normalized to 1, and the relative expression levels at 2 and 12 h were calculated respectively based on 2^−ΔΔCT^ method. In addition, the primers are displayed in **Table 1**.

**Table 1:** Primers.

Gene	The primer sequence (5′-3′)
OGA	Forward: AGCCAAATGGTGACAAGGAACTCTC
	Reverse: GCTCCGACCAAGTATAACCACATCC
β-actin	Forward: ACGGCCAGGTCATCACCATT
	Reverse: CGGAGTACTTGCGCTCAGG

### Single-Cell Transcriptome Sequencing and Data Analysis

According to the manufacturer’s instructions, we used the Single-Cell Full-Length mRNA-Amplification Kit (Vazyme, n712) to extract and reversely transcribe the total RNA of oocytes. Each group had five oocytes. The cDNA products were purified by VAHTS DNA Clean Beads (Vazyme, N411). The amount and purity of cDNA were evaluated by Qubit (Invitrogen, USA) and Bioanalyzer 2100 (Agilent, USA). Finally, we used the TruePrep DNA Library Prep Kit V2 for Illumina (Vazyme, TD502) to prepare RNA libraries. Furthermore, Illumina Novaseq™ 6000 (LC Bio Technology CO., Ltd. Hangzhou, China) was used to conduct pair-end sequencing according to the standard operation protocol, with the sequencing mode of PE150. The quality control of FASTQ files was performed by Trim Galore software. The adaptors, low-quality sequences with the default parameters, and repeated sequences were removed. The paired-end clean reads were mapped to the reference genome of Mus musculus GRCh38 using HISAT2 (25). We used the feature Counts function of subread software to analyze gene quantification (26). In addition, the differential expression analysis was conducted by DESeq2 R package (27). The count value of gene expression was standardized to transcripts per kilobase of exon model per million mapped reads value. The genes with fold change >1.5 or < 0.7 and p-value < 0.05 were considered differentially expressed genes (DEGs).

### Statistical Analysis

SPSS 25.0 (SPSS Inc., IL, USA) was used for statistical analyses, and GraphPad Prism 8 (GraphPad Software, CA, USA) was conducted for graph visualization. Data are presented as the mean ± standard error of mean (SEM). Comparison between two groups was analyzed by Student’s t-test (two-tailed). Results were considered statistically significant when *P* < 0.05(*P < 0.05, **P < 0.01, and ***P < 0.001).

## Results

### Expression Profiling of NAT10-Depleted Mouse Oocytes

**Figure 1:**
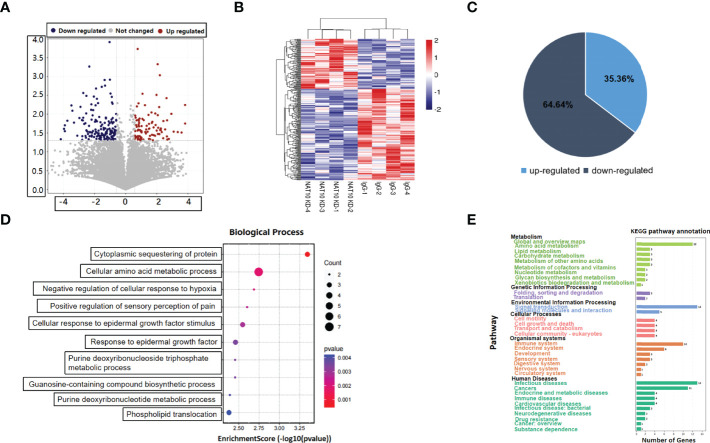
Expression profiling of NAT10-depleted mouse oocytes. **(A)**Volcano map showed the gene expression with NAT10 KD. Blue dots represented downregulation, and red dots represented upregulation. **(B)** The heatmap showed clusters of differential expression of genes. **(C)** Pie chart presented the proportion of up/downregulated genes in 280 DEGs. **(D)** Cellular amino acid metabolic process and cytoplasmic sequestering of protein were main biological process of 280 DEGs. **(E)** A total of 280 DEGs were enriched in metabolism and signal transduction according to KEGG enrichment analysis.

We have previously reported that NAT10 depletion resulted in retarded meiotic progression in mouse oocytes, and NAT10-mediated ac4C modification was a crucial regulator during oocyte maturation (20). In the current study, we knocked down NAT10 in GV stage mouse oocytes and performed transcriptome analysis to further identify the regulated genes and investigated the underlying mechanisms. As shown in **Figure 1A**, the volcano plot demonstrated the DEGs between NAT10-depleted and control oocytes. Clusters of differential expression were shown by the heatmap, and most of the genes were downregulated with NAT10 depletion (**Figure 1B**). Bioinformatic analysis identified 280 DEGs with NAT10 KD, including 99 upregulated genes and 181 downregulated genes, implying that NAT10 KD resulted in enhanced degradation of transcripts (**Figure 1C**). All of the 280 identified DEGs were subjected to GO enrichment and KEGG pathway analysis to better understand their biological functions. These genes were mainly enriched in the biological processes related to cellular amino acid metabolic process and cytoplasmic sequestering of protein (**Figure 1D**). Analysis of KEGG pathway showed that DEGs were enriched in the metabolism and signal transduction (**Figure 1E**).

### Transcripts Modulated by NAT10-Mediated ac4C Modification in Oocytes

**Figure 2:**
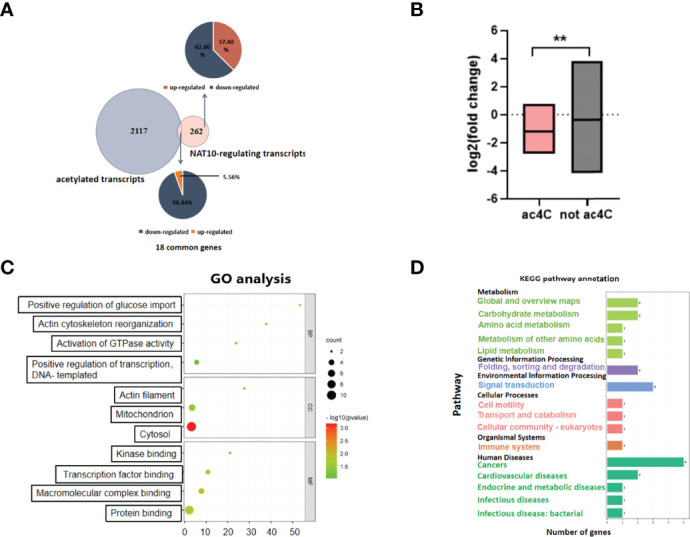
Transcripts modulated by NAT10-mediated ac4C modification in oocytes. **(A)** Venn diagram and pie chart showed the proportion of NAT10-regulated ac4C genes and non–ac4C-modified genes. **(B)** Box diagram displayed the fold change difference between NAT10-regulated ac4C-modified DEGs and non–ac4C-modified DEGs. **(C)** Sixteen downregulated ac4C-modified DEGs regulated by NAT10 mainly participated in regulation of protein binding and positive regulation of transcription, DNA-templated. **(D)** KEGG analysis showed 16 downregulated ac4C-modified DEGs regulated by NAT10 were enriched in cancer, signal transduction, folding, sorting, and degradation. Unpaired t-test was used in **(B)** to compare the expression of genes because the number of genes did not match between two groups. **P < 0.01.

To identify the genes modulated by NAT10-mediated ac4C modification, we obtained 2,135 genes that have been reported to be acetylated in a previous study (28). There were 18 transcripts with potential ac4C sites among the 280 DEGs. Among them, as many as 17 genes were downregulated with NAT10 KD. Given that ac4C has been shown to enhance mRNA stability, these findings suggested that NAT10 might modulate gene expression in an ac4C-dependent manner (16, 28). As for the 262 NAT10-modulated genes without potential ac4C sites, 62.60% were downregulated and 37.40% were upregulated. NAT10 might have an indirect effect on these genes (**Figure 2A**). With NAT10 depletion, the overall expression of genes with and without potential ac4C sites were both downregulated, but the decline of potentially acetylated genes was more pronounced (**Figure 2B**). These data were consistent with the known functions of ac4C, implying that NAT10 exerts its function on downstream genes mainly through ac4C modification. GO enrichment and KEGG pathway analyses were conducted for the downregulated ac4C transcripts. *GPR153* was not included in the analyses because of a fold change of 0. Functional annotation showed that these transcripts were enriched in biological functions as regulation of protein binding and positive regulation of transcription, DNA-templated (**Figure 2C**). In addition, they were enriched in pathways associated with cancer, signal transduction, folding, sorting, and degradation (**Figure 2D**).

### *OGA* Stability Was Modulated by NAT10-Mediated ac4C Modification

Because NAT10-mediated ac4C modification was demonstrated to participate in post-transcriptional regulation of mouse oocyte maturation *in vitro* (20), we intended to identify that the NAT10-targeted acetylated transcripts might play a role in modulating IVM. Thus, we further investigated the 18 DEGs with potential ac4C site, among which the abundance of *OGA* was high (>1), and it showed the lowest *P*-value and highest absolute value of fold change. In mammals, OGA is the enzyme to catalyze the removal of O-GlcNAc of proteins (29). O-GlcNAc is recognized as an important regulatory mechanism of cytosolic and nuclear proteins (30). The involvement of O-GlcNAc in regulating mammalian oocyte maturation has been determined in previous literature (31, 32). Frank and colleagues have reported that *in vitro* developmental competence of mouse oocytes was impaired by O-GlcNAc of heat shock protein 90 under hyperglycemic conditions (32). Another study noted that disruption of O-GlcNAc homeostasis during mammalian oocyte meiotic maturation impacted fertilization (31). Therefore, we further explored the regulatory mechanisms of *OGA* as well as its function in IVM modulation.

**Figure 3:**
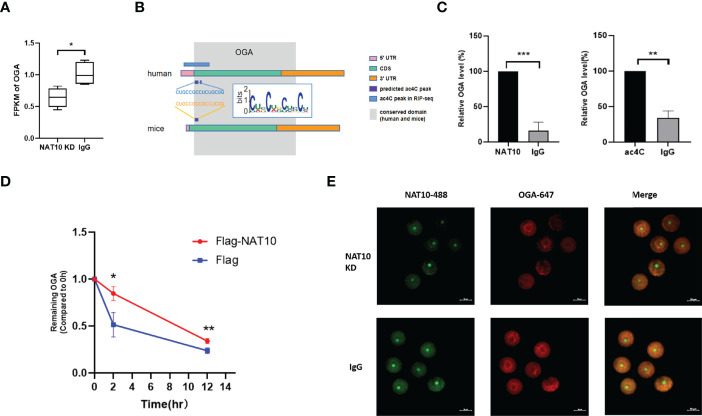
*OGA* Stability was modulated by NAT10-mediated ac4C modification. **(A)** Expression of *OGA* was downregulated significantly by comparing NAT10 KD transcriptome with the control. **(B)** The diagram displayed ac4C-modified potential sites of *OGA* in homo sapiens and Mus musculus. **(C)** Bar chart showed *OGA* could bind to NAT10 and be ac4C-modified by RIP. **(D)** The degradation of *OGA* was suppressed in NAT10-overexpressing compared with negative control group. **(E)** Immunofluorescence showed NAT10 degradation and OGA downregulation after NAT10 KD by Trim-Away. Data represent the mean ± SEM of at least three independent experiments. *P < 0.05, **P < 0.01, and ***P< 0.001.

Our data demonstrated that NAT10 KD resulted in significant downregulation of *OGA* (**Figure 3A**). According to the literature, the acetylated site of *OGA* from Hela cells is located within 484–498 (28). We investigated the sequences of *OGA* from murine and human origins and found that a region of human *OGA* (265–279) is highly conserved with the murine ac4C site (**Figure 3B**). By performing NAT10 RIP and ac4C RIP, we confirmed that *OGA* was modulated by both NAT10 (*P* < 0.001) and ac4C modification (*P* < 0.01) (**Figure 3C**). Because NAT10 is the only known mRNA acetyltransferase in mammals and mainly regulates gene expression in an ac4C-dependant way (28), we inferred that NAT10 regulated *OGA* gene expression by altering ac4C modification. It is well known that transcription ceases and mRNA decay are fundamental events during mammalian oocyte maturation. Oocyte meiosis triggers instability of a subset of mRNAs, leading to active degradation of approximately 20% of accumulated maternal transcripts (33, 34). Because ac4C is known to stabilize mRNA, the degradation of *OGA* was also measured in our study. The results showed that the degradation of *OGA* mRNA was markedly inhibited by NAT10 overexpression (**Figure 3D**). Immunostaining of oocytes showed that NAT10 KD led to decreased expression of *OGA*, further confirming that *OGA* was modulated by NAT10, possibly through an ac4C-dependent way (**Figure 3E**). These results indicated that NAT10-mediated ac4C modulated the expression of *OGA* by inhibiting its decay. Because the modulation of OGA expression level would result in a compensatory change of OGT, we performed immunofluorescence of OGT in oocytes after NAT10 KD to verify the compensatory regulation in our experiment (35). In addition, the results showed that OGA downregulation after NAT10 KD would lead to the decrease of OGT level to maintain the O-GlcNAc homeostasis (**Figure S1**).

### OGA Depletion Retarded Mouse Oocyte Maturation *In Vitro*

**Figure 4:**
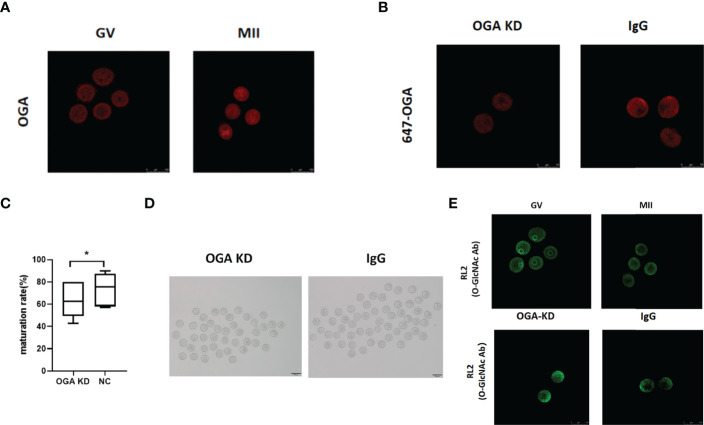
*OGA* depletion retarded mouse oocyte maturation *in vitro.* **(A)** Compared with GV oocytes, *OGA* gene expression was upregulated in MII oocytes. **(B)** OGA was significantly downregulated by Trim-Away. **(C)** Box plot showed the difference of oocyte maturation rates between OGA KD group and the control group. **(D)** Representative pictures of oocyte maturation in OGA KD group and the control group. **(E)** Different expression of O-GlcNAc in GV and MII oocytes was verified by immunofluorescence, and O-GlcNAc modification was upregulated after OGA intervention. Data represent the mean ± SEM of at least three independent experiments. *P < 0.05.

Although O-GlcNAc modification is known as a regulatory mechanism during mammalian oocyte development (31, 32), the role of *OGA* in oocyte maturation remains unclarified. Thus, GV-stage and mouse oocytes maturated *in vitro* were collected for immunostaining. The results revealed significantly increased expression of *OGA* from GV to MII oocytes (**Figure 4A**). To further investigate the role of *OGA* in modulating oocyte maturation, OGA in GV oocytes was knocked down through electroporation, and the effective depletion of OGA was confirmed by immunofluorescence staining (**Figure 4B**). The intervened GV oocytes were cultured in IVM medium for 14–16 h. Interestingly, the *in vitro* maturation rate was significantly reduced with OGA KD (*P* < 0.05) (**Figures 4C, D**). In mammals, OGA is recognized as the critical enzyme to remove O-GlcNAc of proteins (36). We further investigated the role of O-GlcNAc in oocyte maturation. During mouse oocyte meiotic progression, markedly decreased level of O-GlcNAc was observed. In addition, with OGA depletion, impaired meiotic maturation was accompanied with enhanced level of O-GlcNAc (**Figure 4E**). Collectively, OGA depletion led to impaired meiotic progression of mouse oocytes, possibly *via* enhancing O-GlcNAc of proteins. These results indicated that OGA-modulated O-GlcNAc of proteins acted as a critical regulatory mechanism in oocyte maturation.

### Expression Profiling of Oocytes With OGA Knockdown

**Figure 5:**
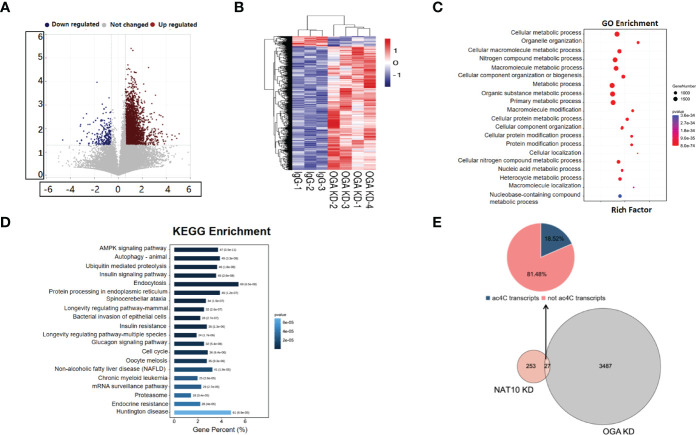
Expression Profiling of Oocytes with OGA Knockdown. **(A)** Volcano map showed global genes change after OGA KD, with blue dots representing downregulation and red dots representing upregulation. **(B)** Heatmap showed the overall clusters of differential expression of genes. **(C)** A total of 3,514 DEGs with OGA KD were mainly associated with metabolic processes according to GO enrichment. **(D)** KEGG analysis displayed top 20 pathways where 3,514 DEGs were involved in. **(E)** Pie chart showed the distribution of DEGs by joint analysis of OGA KD, NAT10-KD transcriptomes, and ac4C RIP data.

*OGA* has been identified as the gene modulated by NAT10-mediated ac4C, which played an important role in oocyte maturation. We depleted OGA in GV stage oocytes and performed transcriptome analysis to further unravel the altered genes and the possible mechanisms. The volcano plot showed the differential expressed genes between OGA-depleted and control oocytes (**Figure 5A**). As demonstrated in **Figure 5B**, most genes were upregulated with OGA KD, possibly associated with an elevated level of O-GlcNAc modification. GO enrichment analysis confirmed that the differentially expressed transcripts were associated with metabolic processes (**Figure 5C**). Pathway analysis was also performed on the basis of KEGG database and the most significant pathways were displayed in **Figure 5D**, including cell cycle and oocyte meiosis. To investigate the downstream mechanisms by which NAT10 regulated OGA through ac4C modification and thus modulated oocyte maturation, the expression profiling with OGA KD, NAT10-depleted transcriptome, and ac4C RIP data was analyzed jointly (28). A total of 27 DEGs were identified in common, out of which 22 genes were not ac4C-modified (**Figure 5E**). The altered expression of these 22 genes cannot be mediated directly by ac4C. Instead, these genes might be modulated by OGA, which could be modified by NAT10, too.

### Joint Analysis of NAT10-Depleted and OGA-Depleted Transcriptomes

**Figure 6:**
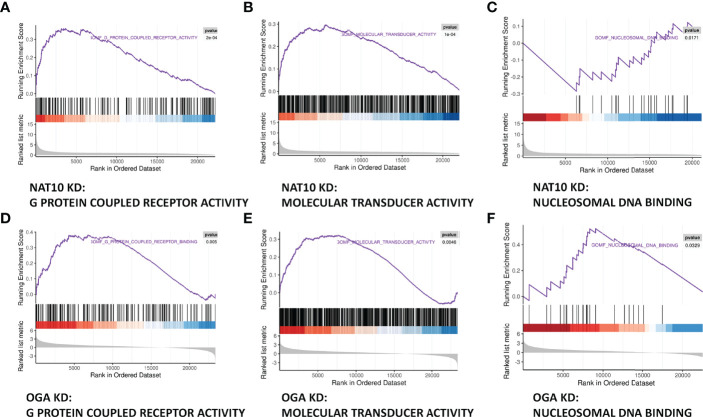
Joint Analysis of NAT10-depleted and OGA-depleted Transcriptomes. **(A–C)** GSEA analysis showed that NAT10 KD transcriptome genes were mainly enriched in G protein–coupled receptor activity, molecular transducer activity, and nucleosomal DNA binding. **(D–F)** OGA-depleted expression profiling was enriched in similar gene sets as NAT10-KD according to GSEA analysis.

**Figure 7:**
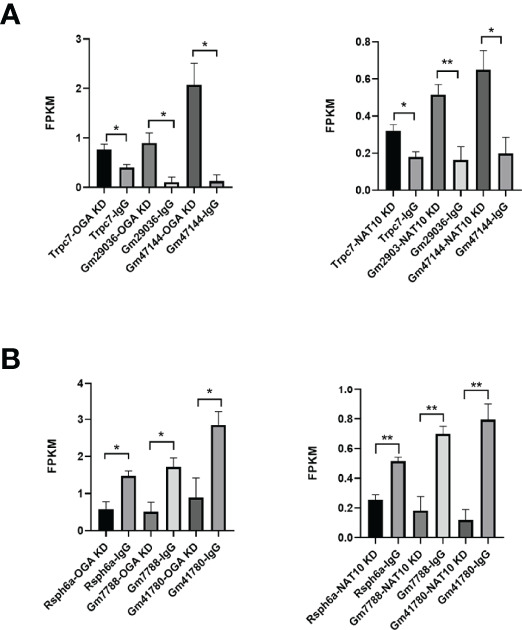
Co-regulated genes of NAT10 and OGA in oocyte maturation. **(A)** Common upregulated DEGs and their expression in NAT10 KD and OGA KD transcriptomes. **(B)** Common downregulated DEGs and expression in NAT10 KD and OGA KD transcriptomes. *P < 0.05 and **P < 0.01.

Gene set enrichment analyses (GSEA) of NAT10-depleted and OGA-depleted transcriptomes were further performed to gain insights about the biological significance of DEGs. The results suggested that NAT10 was associated with G protein–coupled receptor activity, molecular transducer activity, and nucleosomal DNA binding (**Figures 6A–C**). Interestingly, the OGA-depleted expression profiling was enriched in similar gene sets (**Figures 6D–F**). Taken together, NAT10 might affect these aforementioned biological processes by regulating ac4C modification of *OGA* mRNA and thus participate in the regulation of oocyte maturation. We next investigated each DEG and identified several genes co-regulated by NAT10 and OGA. As shown in **Figure 7A**, the expression of *Rsph6a*, *Gm7788*, and *Gm41780* was downregulated in both NAT10-depleted and OGA-depleted oocytes. Among them, *Rsph6a* is associated with mammalian fertility and is recognized as a potential marker for fertility (37). In addition,*Trpc7*, *Gm29036*, and *Gm47144* were upregulated in both NAT10-silenced and OGA-silenced groups (**Figure 7B**). Among them, *Trpc7* is involved in the regulation of calcium ion transmembrane transport and cytoplasmic calcium ion concentration, which are critical for oocyte maturation and activation (38, 39). *Rsph6a* and *Trpc7* were possible downstream genes modulated by NAT10-mediated ac4C on *OGA* mRNA, and their dysregulation could result in impaired oocyte maturation.

## Discussion

The purpose of this study is to explore the important downstream genes and mechanisms of NAT10-mediated ac4C modification during oocyte maturation. In a previous study, we found that ac4C downregulation after NAT10 KD inhibited oocyte maturation, but the underlying mechanisms remained unknown (20). To further explore the underlying mechanisms, we conducted a transcriptomic study on oocytes with NAT10 KD and analyzed it with ac4C RIP data jointly (28). Our result revealed that *OGA* was one of the important target genes of NAT10-mediated ac4C modification during oocyte maturation.

First, *OG*A was verified to bind to NAT10 and be ac4C-modified based on NAT10/ac4C RIP results. Furthermore, to explore the function of NAT10-mediated ac4C modification on *OGA*, we conducted degradation experiments and found that NAT10 might regulate the expression of *OGA* by suppressing the degradation of it.

We furthered on investigating whether NAT10-mediated ac4C modification affected oocyte maturation through *OGA*. As the role of *OGA* in mammalian oocyte maturation has not been reported yet, we compared the fluorescent expression of *OGA* in GV and MII oocytes, and the latter was significantly higher than the former. *OGA* seemed to be a beneficial factor for oocyte maturation, whereas the maturation was impaired after OGA KD compared with the control group. According to the previous studies, OGA is a key protein in O-GlcNAc modification, which regulates the progression of cell cycle, cell signal transduction, and mitochondrial function by removing O-GlcNAc from the target proteins (40). Thus, we further explored the O-GlcNAc fluctuation by comparing RL2 expression between GV and MII oocytes, and interestingly, as oocytes maturated, the O-GlcNAc modification decreased significantly, which indicated the importance of OGA regulating O-GlcNAc level during IVM. On the basis of the above results, it is reasonable to speculate that *OGA* was the important regulated gene of NAT10-mediated ac4C modification in IVM.

To further explore the role and mechanisms of NAT10-mediated *OGA* ac4C in oocyte maturation, we knocked down *OGA* of oocytes for transcriptome sequencing. In addition, through joint analysis with the transcriptome data of oocytes depleted NAT10, we found that NAT10-mediated *OGA* ac4C regulated oocyte maturation mainly through G protein–coupled receptor, molecular transducer activity, and nucleosomal DNA binding. *Rsph6a*, *Gm7788*, and *Gm41780* were downregulated in NAT10-depleted and OGA-depleted transcriptomes, whereas *Trpc7*, *Gm29036*, and *Gm47144* were upregulated. As *Rsph6a and Trpc7* played an important part in fertility, we speculated that both of them might be the important downstream genes in NAT10-mediated *OGA* ac4C modification in oocyte maturation.

Previous literatures have reported the function of O-GlcNAc modification in oocyte development and maturation (31, 32, 41–43). Slawson et al. found that glucosamine or PUGNAc treatment impaired the maturation kinetics of Xenopus laevis oocytes because the O-GlcNAc level increased, and oocytes at advanced stages (III–IV) were less O-GlcNAc–modified compared with that of stages I–II (44). Dehennaut et al. got the similar results and identified that OGA activity attributed to the decreased O-GlcNAc level as oocytes maturated in Xenopus laevis but not decreased OGT expression nor the decrease of the substrate (42). It supports our finding that OGA KD resulted in elevated O-GlcNAc modification and thus jeopardized oocyte development potential. It is worthy to note that most studies about O-GlcNAcylation in oocytes were carried out in the non-mammal such as Xenopus because of the technology limit and the available number of oocytes (45). We performed it in mice oocytes, which might be more consistent with the maturation progress of human oocytes.

Because oocyte maturation is a finely regulated process, epigenetic modifications play a crucial role to ensure timely and selective translation or degradation of specific RNAs (46). Sequencing and omic techniques should be emphasized in exploring the specific characterization and key events in oocyte maturation (8). Our study combined two transcriptome data and proved that *OGA* was regulated by NAT10-mediated ac4C modification, which might affect oocyte maturation through G protein–coupled receptors, molecular transduction, and nucleosome DNA binding. It will also provide insights for the modification interaction between ac4C modification and O-GlcNAcylation. What is noteworthy is that both of the two epigenetic modifications are new and that there are few studies on the role and mechanisms of them in mammalian oocyte maturation, so this is the main innovation of our research.

In conclusion, our results demonstrated that NAT10 might stabilize *OGA* through ac4C modification and mediated oocyte maturation. In addition, our results suggested that NAT10 might affect O-GlcNAc level by regulating OGA, thus regulating key proteins that could be O-GlcNAc–modified in IVM. Therefore, our study further explored the mechanisms of epigenetic modification in oocyte maturation and would be used as reference for clinical improvement of IVM.

## Data Availability Statement

The data presented in the study are deposited in the GSA repository (https://bigd.big.ac.cn/gsa/browse/CRA007092, accession number CRA007092).

## Ethics Statement

Animal experiments were approved by the Animal Care and Use Committee of the Sixth Affiliated Hospital, Sun Yat-sen University (Guangzhou, China) (ethical approval number: IACUC-2021112502).

## Author Contributions

CZ and JLi designed the study. XL supervised the progress of it and provided the funding. JLin performed the experiment with the help of HZ, YZ, JLin, QL, and TW. JLin drafted the manuscript with the support of YX and JH. All authors contributed to the article and approved the submitted version.

## Funding

This study was supported by the National Key Research and Development Program of China (2021YFC2700403), National Natural Science Foundation of China (82071713), Natural Science Foundation of Guangdong Province (2019A1515011764), and Youth Project-Joint Foundation for Basic and Applied Basic Research of Guangdong Province (2021A1515110977).

## Conflict of Interest

The authors declare that the research was conducted in the absence of any commercial or financial relationships that could be construed as a potential conflict of interest.

## Publisher’s Note

All claims expressed in this article are solely those of the authors and do not necessarily represent those of their affiliated organizations, or those of the publisher, the editors and the reviewers. Any product that may be evaluated in this article, or claim that may be made by its manufacturer, is not guaranteed or endorsed by the publisher.

## Supplementary Material

Supplementary Figure 1 The modulation of OGA level after NAT10 knockdown induced a compensatory regulation of OGT. Affected by NAT10 knockdown, the decreased level of OGA resulted in the downregulation of OGT in oocytes.
