# Supplementary figures and images for: NAT10 Maintains OGA mRNA Stability Through ac4C Modification in Regulating Oocyte Maturation
Source: Front Endocrinol (Lausanne). 2022 Jul 22;13:907286. doi: 10.3389/fendo.2022.907286 (PMC9352860; doi:10.3389/fendo.2022.907286)

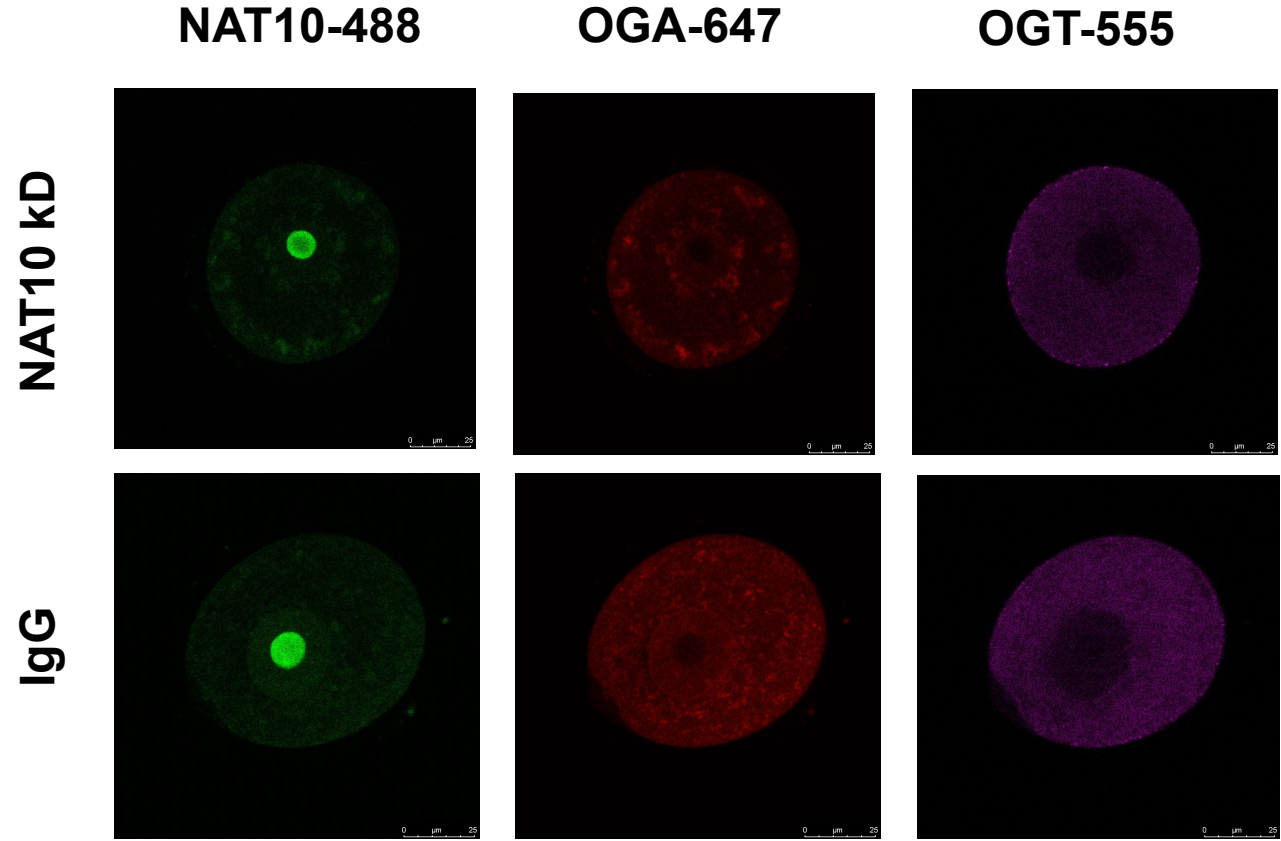

Supplement: Supplementary Figure 1 — The modulation of OGA level after NAT10 knockdown induced a compensatory regulation of OGT. Affected by NAT10 knockdown, the decreased level of OGA resulted in the downregulation of OGT in oocytes. [file Image_1.pdf]
